# Multicentre analysis of PET SUV using vendor-neutral software: the Japanese Harmonization Technology (J-Hart) study

**DOI:** 10.1186/s13550-018-0438-9

**Published:** 2018-08-20

**Authors:** Yuji Tsutsui, Hiromitsu Daisaki, Go Akamatsu, Takuro Umeda, Matsuyoshi Ogawa, Hironori Kajiwara, Shigeto Kawase, Minoru Sakurai, Hiroyuki Nishida, Keiichi Magota, Kazuaki Mori, Masayuki Sasaki, J-Hart study group

**Affiliations:** 1 Division of Radiology, Department of Medical Technology, Kyushu University Hospital, 3-1-1 Maidashi, Higashi-ku, Fukuoka, 812-8582 Japan; 2 Gunma Prefectural College of Health Sciences, 323-1 Kamioki-machi, Maebashi-shi, 371-0052 Japan; 3 National Institute of Radiological Sciences, National Institutes for Quantum and Radiological Science and Technology, 4-9-1 Anagawa, Inage-ku, Chiba-shi, 263-8555 Japan; 4 Department of Molecular Imaging, Institute of Biomedical Research and Innovation, 2-2, Minatojima Minamimachi, Chuo-ku, Tokyo, 28 650-0047 Japan; 5 Department of Nuclear Medicine, Cancer Institute Hospital of Japanese Foundation for Cancer Research, 3-8-31 Ariake, Koto-ku, Tokyo, 135-8550 Japan; 6 Department of Radiology, Yokohama City University, 3-9 Fukuura, Kanazawa-ku, Yokohama, 236-0004 Japan; 7 Department of Radiology, Center Hospital of National Center for Global Health and Medicine, 1-21-1 Toyama Shinjuku-ku, Tokyo, 162-8655 Japan; 8 Department of Radiology, Kyoto University Hospital, 54 Kawaharacho, Syogoin, Sakyo-ku, Kyoto City, 606-8507 Japan; 9 Clinical Imaging Center for Healthcare, Nippon Medical School, 1-12-15 Sendagi, Bunkyo-ku, Tokyo, 113-0022 Japan; 10 Division of Medical Imaging and Technology, Hokkaido University Hospital, Kita 14-jo Nishi 5-chome, Kita-ku, Sapporo-shi, Hokkaido 060-8648 Japan; 11 Department of Radiology, Toranomon Hospital, 2-2-2 Toranomon, Minato-ku, Tokyo, 105-8470 Japan; 12 Department of Health Science, Faculty of Medical Sciences, Kyushu University, 3-1-1 Maidashi, Higashi-ku, Fukuoka, 812-8582 Japan

**Keywords:** FDG PET/CT, SUV, Harmonization, Multicentre study

## Abstract

**Background:**

Recent developments in hardware and software for PET technologies have resulted in wide variations in basic performance. Multicentre studies require a standard imaging protocol and SUV harmonization to reduce inter- and intra-scanner variability in the SUV. The Japanese standardised uptake value (SUV) Harmonization Technology (J-Hart) study aimed to determine the applicability of vendor-neutral software on the SUV derived from positron emission tomography (PET) images. The effects of SUV harmonization were evaluated based on the reproducibility of several scanners and the repeatability of an individual scanner.

Images were acquired from 12 PET scanners at nine institutions. PET images were acquired over a period of 30 min from a National Electrical Manufacturers Association (NEMA) International Electrotechnical Commission (IEC) body phantom containing six spheres of different diameters and an ^18^F solution with a background activity of 2.65 kBq/mL and a sphere-to-background ratio of 4. The images were reconstructed to determine parameters for harmonization and to evaluate reproducibility. PET images with 2-min acquisition × 15 contiguous frames were reconstructed to evaluate repeatability. Various Gaussian filters (GFs) with full-width at half maximum (FWHM) values ranging from 1 to 15 mm in 1-mm increments were also applied using vendor-neutral software. The SUV_max_ of spheres was compared with the reference range proposed by the Japanese Society of Nuclear Medicine (JSNM) and the digital reference object (DRO) of the NEMA phantom. The coefficient of variation (CV) of the SUV_max_ determined using 12 PET scanners (CV_repro_) was measured to evaluate reproducibility. The CV of the SUV_max_ determined from 15 frames (CV_repeat_) per PET scanner was measured to determine repeatability.

**Results:**

Three PET scanners did not require an additional GF for harmonization, whereas the other nine required additional FWHM values of GF ranging from 5 to 9 mm. The pre- and post-harmonization CV_repro_ of six spheres were (means ± SD) 9.45% ± 4.69% (range, 3.83–15.3%) and 6.05% ± 3.61% (range, 2.30–10.7%), respectively. Harmonization significantly improved reproducibility of PET SUV_max_ (*P* = 0.0055). The pre- and post-harmonization CV_repeat_ of nine scanners were (means ± SD) 6.59% ± 1.29% (range, 5.00–8.98%) and 4.88% ± 1.64% (range, 2.65–6.72%), respectively. Harmonization also significantly improved the repeatability of PET SUV_max_ (*P* < 0.0001).

**Conclusions:**

Harmonizing SUV using vendor-neutral software produced SUV_max_ for 12 scanners that fell within the JSNM reference range of a NEMA body phantom and improved SUV_max_ reproducibility and repeatability.

## Background

^18^F-fluoro-2-deoxy-2-d-glucose (^18^F-FDG) positron emission tomography (PET) is a valuable imaging tool for the diagnosis, staging and assessment of the responses of various malignancies to therapy [1–5]. Reports indicate that ^18^F-FDG PET is a more effective biomarker of treatment responses than morphological information because it measures glucose metabolism [6–9]. The standardised uptake value (SUV) has served as a semi-quantitative metric of ^18^F-FDG uptake by tumours.

The SUV must be highly reproducible and repeatable to serve as a reliable biomarker in multicentre trials using various PET scanners, because SUV variability among scanners can contribute to uncertainty in results. The Quantitative Imaging Biomarker Alliance (QIBA), which was organised by the Radiological Society of North America (RSNA), recommended definitions for reproducibility and repeatability [10, 11]. In short, reproducibility refers to the consistency of values derived from repeated tests of one individual by different operators, different scanners, software, or at different sites and times. Repeatability refers to the consistency of values derived from repeated tests of one individual by a single operator using the same scanner and software. That is, reproducibility refers to inter-scanner variability, whereas repeatability refers to intra-scanner variability.

Recent developments in hardware and software for PET technologies have resulted in wide variations in basic performance. The SUV considerably varies due to biological and technical factors involving the model of the PET scanner, acquisition protocols, reconstruction algorithm and parameters [12–15]. For example, ordered subset expectation maximization (OSEM) with a point spread function (PSF) algorithm improves the spatial resolution, but it causes edge artifacts as overestimation [16, 17]. Therefore, multicentre studies require a standard imaging protocol and SUV harmonization to reduce inter- and intra-scanner variability in the SUV.

Several organizations have published guidelines to standardise ^18^F-FDG PET imaging protocols and harmonize SUV harmonization [14, 18–20]. In terms of SUV harmonization, the European Association of Nuclear Medicine (EANM) and the Japanese Society of Nuclear Medicine (JSNM) have proposed a specified range of recovery coefficients (RC) as a function of sphere size [21]. “Harmonization” is defined herein as a PET image smoothed with an additional Gaussian filter (GF) so that the RC of each sphere falls within the specified range of the body phantom. This harmonization strategy minimises variations in SUV measurements during scan acquisition and processing [22–24] and reduces reconstruction-dependent variability in the PET Response Criteria in Solid Tumours (PERCIST) classification [25].

The usefulness of commercially available software for harmonization was first determined using EQ.PET software [23]. However, a costly, software-specific workstation provided by the Siemens Healthineers is required. The recently commercially available GI-PET software (AZE VirtualPlace Hayabusa, Tokyo, Japan) adjusts the RC of PET images into a reference RC using an additional GF. This vendor-neutral quantitative software can be installed in general personal computers.

We designed the Japanese SUV Harmonization Technology (J-Hart) study based on this background to determine the applicability of vendor-neutral software to multicentre PET SUV harmonization. We also assessed the effects of SUV harmonization on SUV reproducibility and repeatability.

## Methods

### Phantom

We used a NEMA 2001 International Electrotechnical Commission (IEC) body phantom (Data Spectrum Corp., Durham, NC, USA), consisting of six spheres (Model PET/IEC–BODY/P) of 10, 13, 17, 22, 28 and 37 mm in diameter, with a wall thickness of 1 mm in a quasi-cylindrical cavity (280 × 210 × 180 mm). All spheres and the background were filled with solutions containing 10.6 and 2.65 kBq/mL (at the midpoint of 30-min acquisitions), respectively, of ^18^F-FDG to obtain a sphere-to-background ratio of 4.

### PET/CT scanners

Data were acquired at nine Japanese institutions using 12 popular PET systems (Table 1): Discovery ST Elite Performance (DSTEP), Discovery ST Elite (DSTE), Discovery 600 Motion (D600), Discovery 690 (D690) and Discovery IQ (DIQ) scanners (General Electric Medical Systems, Milwaukee, WI, USA); Biograph 64 True Point (BioTP), Biograph mCT 3ring (Bio3R) and Biograph mCT flow 4ring (Bio4R) scanners (Siemens Healthineers, Erlangen, Germany); GEMINI-TF16 (GTF), Gemini GXL (GXL) scanners (Philips Medical Systems, Cleveland, OH, USA); Aquiduo (Aquiduo) and Celesteion PCA-9000A (Celesteion) scanners (Toshiba Medical Systems, Otawara, Japan). The collaborating institutions comprised five university hospitals, three cancer centres and one research institution that normally conduct many oncological PET studies. The dose calibrator and each PET scanner of all evaluated PET/CT systems were cross-calibrated before data acquisition from the phantom according to the guidelines [19].

**Table 1:** Clinical parameters of PET scanners

	Aquiduo (Aquiduo) TOSHIBA	Biograph mCT 3 ring (Bio3R) SIEMENS	Biograph mCT Flow 4 ring (Bio4R) SIEMENS	Biograph 64 True Point (BioTP) SIEMENS	Celesteion PCA-9000A (Celesteion) TOSHIBA	Discovery IQ (DIQ) GE	Discovery ST Elite (DSTE) GE	Discovery STEP (DSTEP) GE	Discovery 600 Motion (D600) GE	Discovery 690 (D690) GE	GEMINI TF16 (GTF) Philips	Gemini GXL (GXL) Philips
Cross-calibration (background SUV)	1.03	1.03	0.96	1.01	1.00	1.04	0.96	1.03	1.04	1.01	1.04	1.03
PET reconstruction												
Reconstruction	FORE OSEM	3D-OSEM PSF TOF	3D-OSEM PSF TOF	3D-OSEM PSF	3D-OSEM	3D-OSEM PSF	3D-OSEM	3D-OSEM	3D-OSEM	3D-OSEM TOF	Full list mode TOF 3D-OSEM	LOR RAMLA
Iterations	4	2	2	2	3	4	2	2	3	3	3	2
Subsets	14	21	21	21	10	12	28	21	16	8	33	–
Smoothing	Gaussian	Gaussian	Gaussian	Gaussian	Gaussian	Gaussian	Gaussian	Gaussian	Gaussian	Gaussian	Smooth/sharp; smooth A	Smooth/sharp; normal
FWHM of filter (mm)	8	6	4	4	6	5	6	5.14	4	4		

*FORE* Fourier Rebinning, *OSEM* ordered subset expectation maximization, *PSF* point spread function, *TOF* time of flight, *LOR* line of response, *RAMLA* row action maximum likelihood algorithm, *FWHM* full-width at half maximum

### Region of interest

The SUV_max_ of the six hot spheres was determined from circular regions of interest (ROIs) placed on the centre slice of images of each sphere. The diameter of each ROI was equal to that of each hot sphere (10, 13, 17, 22, 28 and 37 mm). Twelve circular ROIs with a diameter of 10 mm were placed on the background region at the centre slice of the PET image; the average SUV was calculated from twelve ROIs as the SUV_mean_ of the background. The acceptance criterion for the SUV_mean_ of the background region was 1.00 ± 0.05. Table 1 shows the average SUV_mean_ of the background region after cross-calibration.

### Digital reference object

**Fig. 1:**
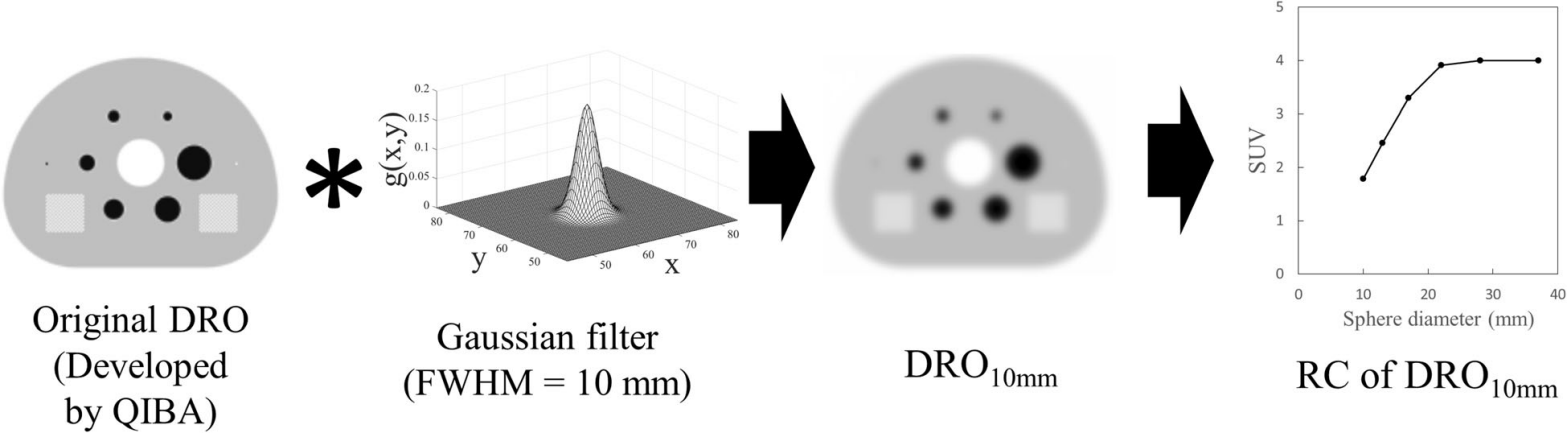
Development of reference recovery coefficient from digital reference object and three-dimensional Gaussian filter

The QIBA suggested that computational imaging models or phantom data might play an important role in reproducibility assessment [26] and thus developed an ^18^F-FDG PET/CT digital reference object (DRO), which is a synthetic test object in Digital Imaging and Communications in Medicine (DICOM) format [27]. We obtained the DRO from the QIBA [28]. The mathematically developed DRO of the NEMA body phantom is an ideal object with a uniform background with a transaxial diameter of 20 cm that simulates a human abdominal cross section with an SUV of 1.00. Six spheres with diameters of 10, 13, 17, 22, 28 and 37 mm contained an ^18^F-FDG solution with an SUV of 4.00. The SUV of a central cylinder with a diameter of 5 cm was 0.00. A three-dimensional (3-D) GF was applied to the DRO to simulate a PET image with relatively low spatial resolution. The RC of the DRO was included in the JSNM reference range for a GF with a full-width at half maximum (FWHM) value of 10–13 mm, and the RC was highest at a 10-mm FWHM of GF (Fig. 1). We adopted DRO_10mm_ as the reference SUV (SUV_ref_) for harmonization.

### Data acquisition and processing

Emission data were acquired from the phantom over a period of 30 min using list mode with the bed in position 1. A PET image with 30-min acquisition was reconstructed to determine the parameters for harmonization and to evaluate reproducibility. PET images with 2-min acquisition × 15 contiguous frames were reconstructed to evaluate the repeatability. The reconstruction parameters were individually determined according to the clinical setting at each institution (Table 1). All reconstructed PET images were output in DICOM format.

### Harmonization

We used GI-PET software on a personal computer for harmonization processing. Stand-alone GI-PET software can quantify PET images and it is typically used to adjust spatial resolution to harmonize PET images using a 3-D GF.

The harmonization procedure of GI-PET was as follows: Firstly, reconstructed PET images with 30-min acquisitions in DICOM format were loaded into the GI-PET software. The original voxels were converted into isotropic voxels of an equivalent size in the *x*, *y* and *z* directions using the bi-linear method because the 3D-GF of GI-PET requires isotropic data. A 3-D GF with various FWHM values of 1–15 mm in 1-mm increments was applied to the PET images, and then, a graph of the SUV_max_ of the spheres was generated as a function of the diameter. The FWHM of the GF that provided an SUV_max_ within the reference range proposed by the JSNM [21] was determined. The reference ranges of the lower to the upper limits of the SUV_max_ on 10-, 13-, 17-, 22-, 28- and 37-mm hot spheres were 1.19–2.00, 1.52–3.04, 2.58–3.71, 3.25–4.09, 3.56–4.21 and 3.82–4.17, respectively. If only one FWHM provided an SUV_max_ within the reference range, then that FWHM was taken as the optimal parameter of GF for harmonization. On the other hand, if several FWHM provided SUV_max_ within the reference range, we compared the root mean square error (RMSE) with DRO_10mm_. The RMSE is the square root of the variance in the SUV_max_ of the six spheres between PET images and DRO_10mm_ and is calculated as:

$$ \mathrm{RMSE}=\sqrt{\frac{1}{6}\sum \limits_{i=10,13,17,22,28,37\ \mathrm{mm}}{\left({\mathrm{SUV}}_{\max\ \mathrm{in}\ \mathrm{PET}\ \mathrm{scanner},i}-{\mathrm{SUV}}_{\max\ \mathrm{in}\ \mathrm{DRO}10\mathrm{mm},i}\right)}^2} $$

**Fig. 2:**
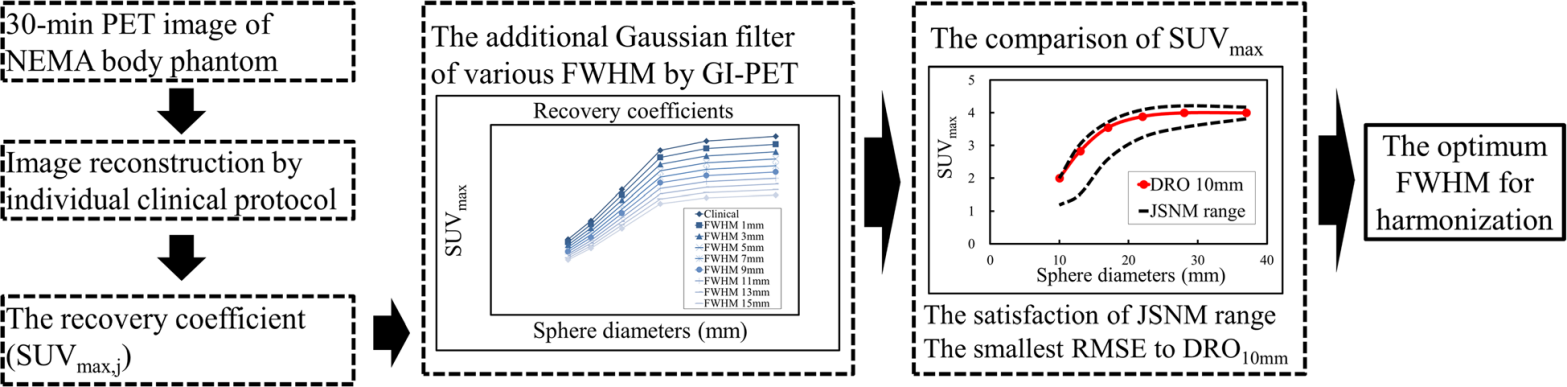
Flow chart used to determine the optimal FWHM of additional Gaussian filter for harmonization

Finally, we determined the optimal FWHM that provided the smallest RMSE (Fig. 2). RSME is a simple mathematical measure that has been used extensively in nuclear medicine and molecular imaging for several years. Combining RSME with DRO is reportedly useful to create appropriate reconstruction conditions for other types of nuclear medicine image [29].

### Reproducibility and repeatability

We calculated the coefficients of variation (CV) of the SUV_max_ on images of spheres with different diameters acquired over a period of 30 min by 12 PET scanners to evaluate the reproducibility of quantitation and defined the CV across the scanners as CV_repro_. We then compared CV_repro_ between the pre- and post-harmonized images calculated as the mean ± standard deviation (SD). The CV is the ratio of the standard deviation (SD) of the mean calculated as:

$$ \mathrm{CV}=\raisebox{1ex}{$\mathrm{SD}$}\!\left/ \!\raisebox{-1ex}{$\mathrm{mean}$}\right.\times 100\ \left(\%\right) $$

We calculated the CV of SUV_max_ on PET images with 2-min acquisitions × contiguous 15 frames for each PET scanner and each sphere size to evaluate the repeatability of quantitation and then defined the CV across the frames as CV_repeat_. We then compared CV_repeat_ between the pre- and post-harmonized images calculated as the mean ± SD.

### Statistical analyses

Differences in CV_repro_ and CV_repeat_ were compared among six and 54 pairs, respectively, between pre- and post-harmonization by Wilcoxon signed-rank tests using JMP® 13 (SAS Institute Inc., Cary, NC, USA). *P* values < 0.05 were considered statistically significant.

## Results

### Harmonization

**Fig. 3:**
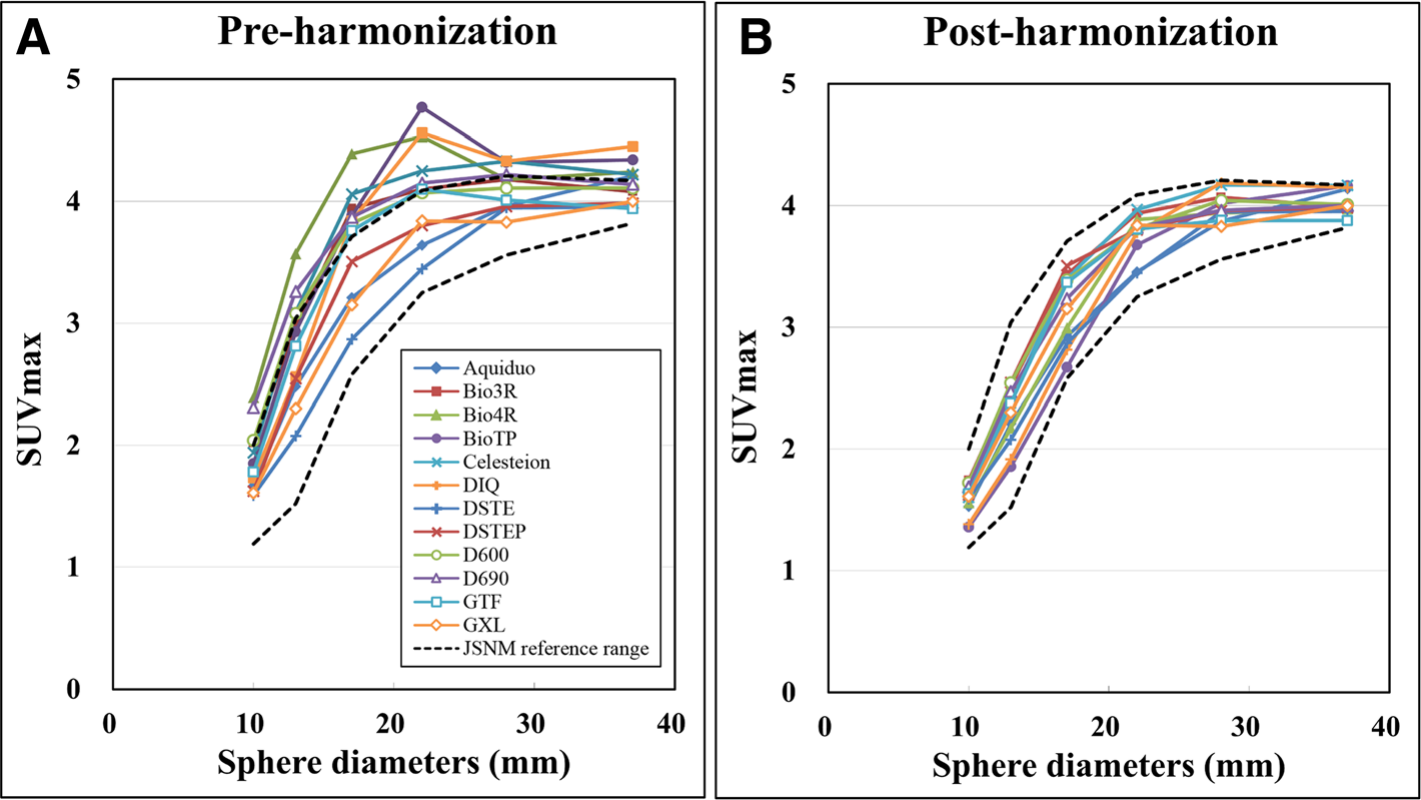
Recovery coefficients of 12 PET scanners obtained from 30-min PET image. Comparison among **a** pre- and **b** post-harmonization RC

The SUV_max_ fell within the JSNM reference range in three PET scanners (DSTE, DSTEP and GXL) without adding a GF (Table 2). Thus, adjustment was not required for harmonization. The FWHM values of the additional GF that resulted in an SUV_max_ within the JSNM reference range were 5–7, 5–9, 8–10, 9, 6–10, 8–9, 6–9, 6–9 and 5–7 mm for the Aquiduo, Bio3R, Bio4R, BioTP, Celesteion, DIQ, D600, D690 and GTF, respectively. The RMSE compared with DRO_10mm_ was the smallest at FWHM values of 5, 5, 8, 9, 6, 8, 6, 6 and 5 mm, respectively (Table 2). The SUV_max_ of these nine scanners fell within the JSNM reference range only after harmonization. Figure 3 shows the pre- and post-harmonization RC of 12 PET scanners.

**Table 2:** Full-width at half maximum for SUV harmonization of PET scanners

PET scanner	Range of FWHM for GF for inclusion in JSNM reference range (mm)	Optimum FWHM for GF to obtain smallest RMSE compared with DRO_10mm_ (mm)
Aquiduo	5–7	5
Bio3R	5–9	5
Bio4R	8–10	8
BioTP	9	9
Celesteion	6–10	6
DIQ	8, 9	8
DSTE	No filter	No filter
DSTEP	No filter	No filter
D600	6–9	6
D690	6–9	6
GTF	5–7	5
GXL	No filter	No filter

*DRO* digital reference object, *FWHM* full-width at half maximum, *GF* Gaussian filter, *RMSE* root mean square error

### Reproducibility

**Fig. 4:**
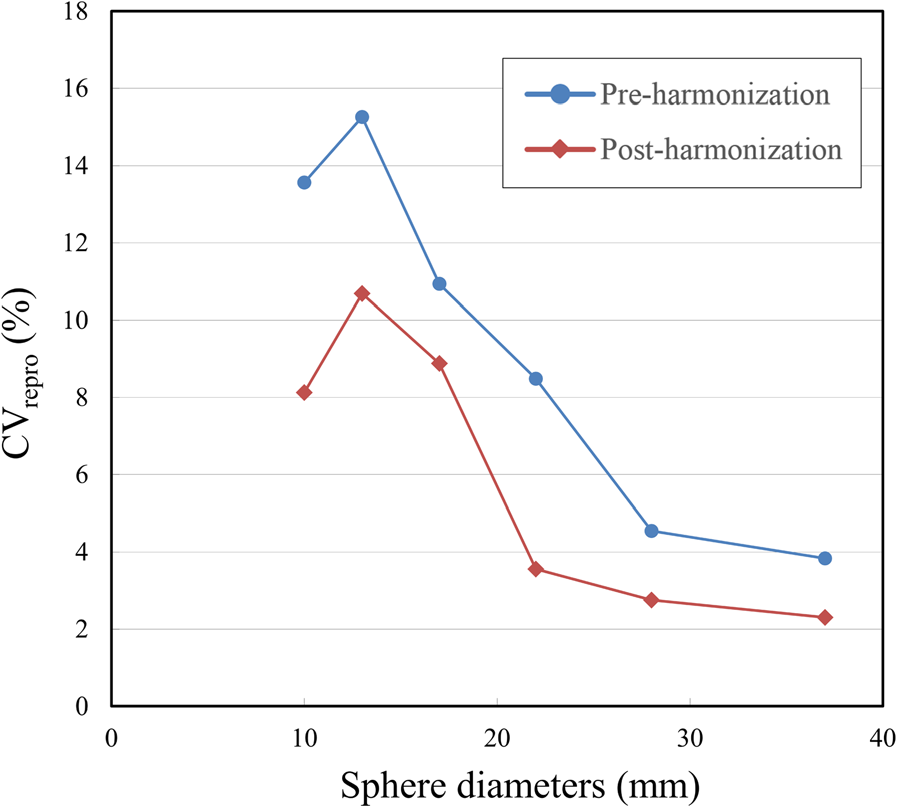
Effects of harmonization on reproducibility. Pre- and post-harmonization comparisons of coefficients of variation of SUV_max_ for 12 PET scanners (CV_repro_)

Figure 4 shows the pre- and post-harmonization CV_repro_ of the 30-min PET images among 12 PET scanners. The (means ± SD) pre- and post-harmonization CV_repro_ of six spheres were 9.45% ± 4.69% (range, 3.83–15.3%) and 6.05% ± 3.61% (range, 2.30–10.7%), respectively. Harmonization significantly improved the reproducibility of the PET SUV_max_ (*P* = 0.0055).

### Repeatability

**Fig. 5:**
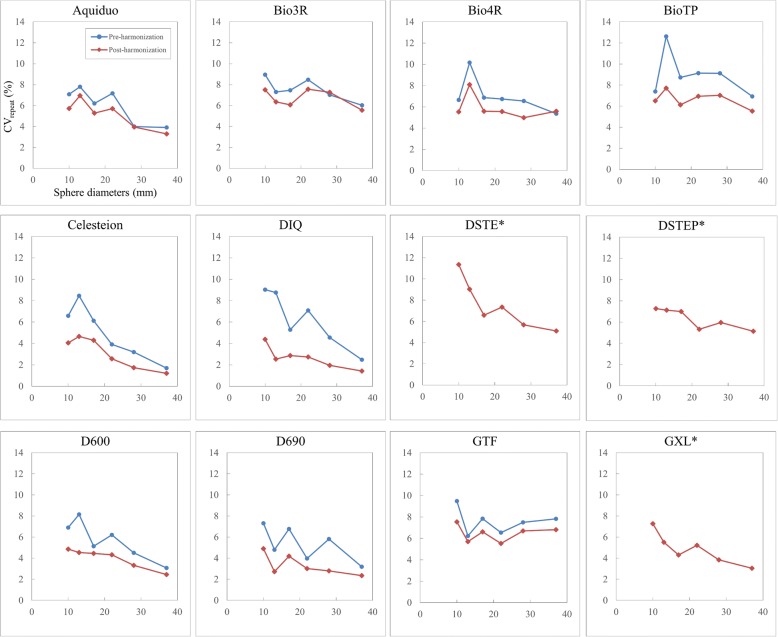
Effects of harmonization on repeatability. Pre- and post-harmonization comparisons of coefficients of variation of the SUV_max_ for 15 frames (CV_repeat_). The asterisk symbol indicates three PET scanners (DSTE, DSTEP and GXL) did not require additional Gaussian filter for harmonization

Figure 5 shows the CV_repeat_ of images across 15 frames acquired from each PET scanner. The post-harmonization CV_repeat_ of the nine PET scanners that required an additional GF for harmonization (Aquiduo, Bio3R, Bio4R, BioTP, Celesteion, DIQ, D600, D690 and GTF) was lower than that at pre-harmonization (Table 3). The pre- and post-harmonization CV_repeat_ (means ± SD) of nine scanners were 6.59% ± 1.29% (range, 5.00–8.98%) and 4.88% ± 1.64% (range, 2.65–6.72%), respectively. Harmonization significantly improved the repeatability of the PET SUV_max_ (*P* < 0.0001).

**Table 3:** Pre- and post-harmonization coefficients of variation across 15 frames of 12 PET scanners

	CV_repeat_ (%)	
	Pre-harmonization	Post-harmonization
Aquiduo	6.03 ± 1.54	5.15 ± 1.21
Bio3R	7.54 ± 0.95	6.72 ± 0.77
Bio4R	7.06 ± 1.48	5.89 ± 1.01
BioTP	8.98 ± 1.83	6.64 ± 0.69
Celesteion	5.00 ± 2.28	3.09 ± 1.32
DIQ	6.20 ± 2.33	2.65 ± 0.92
DSTE	7.51 ± 2.13	–
DSTEP	6.30 ± 0.87	–
D600	5.66 ± 1.65	3.99 ± 0.84
D690	5.31 ± 1.47	3.33 ± 0.90
GTF	7.57 ± 1.06	6.48 ± 0.69
GXL	4.86 ± 1.35	–

*CV* coefficient of variation

## Discussion

The J-Hart study examined whether a vendor-neutral software could harmonize SUV across 12 PET scanners from various manufacturers. We obtained an appropriate FWHM of GF for harmonization by comparing the SUV_max_ of 12 PET scanners at nine institutions with both the JSNM reference range and the DRO_10mm_. Harmonization significantly improved the reproducibility and repeatability of the SUV_max_.

Our harmonization process was based on the simple down-smoothing method proposed by Boellaard et al. [30]. This strategy has been widely applied to SUV harmonization [15, 31] and provides a pair of PET images with relatively high and low resolution. With a comparable SUV across different scanners as well as in EQ.PET software, not only high-resolution images for lesion detection but also harmonized images can be obtained [23]. The EANM provides the strategy for harmonization applied to RC [19], whereas JSNM recommends harmonization applied to SUV_max_ that can detect errors such as cross-calibration. Therefore, SUV_max_ data are presented in graphs of the present study.

Quak et al. validated a proprietary software tool (EQ.PET) to harmonize SUV across three PET systems independently of the applied reconstruction algorithm [23]. They reported that all RC of SUV_max_ fell within EANM curve after applying the EQ.PET filter. The present study confirmed that vendor-neutral GI-PET software could harmonize the SUV_max_ across 12 PET scanners. As a result, all SUV_max_ fell within in the JSNM reference range on GI-PET after adding a GF. Therefore, we consider that GI-PET and EQ.PET are equally useful for harmonization. The post-harmonization SUV_max_ of various PET scanners became comparable based on the same standard, which is relevant to multicentre and follow-up studies that use various PET scanners. E Q.PET is a proprietary software that can only be installed on its own dedicated workstation with PET system. In contrast, GI-PET is a vendor-neutral software that can be applied at any institution using only a personal computer, regardless of the PET system. Although the present study used the JSNM reference range of SUV_max_, the EANM reference range can be used in vendor-neutral software.

Harmonization of the SUV improved the CV_repro_ of SUV_max_ across 12 scanners in the present study. Takahashi et al. reported a maximum difference of 45.7% in the SUV_max_ with a 27-mm sphere across five different PET scanners [32]. Such a substantial variation is a problem for reproducibility. Quak et al. evaluated harmonization across three PET scanners using EQ.PET for 1380 FDG-avid tumours [23]. They reported that the mean ratio with and without PSF of the SUV_max_ for 1380 tumour lesions was 1.46 (95%CI 0.86–2.06) before and 1.02 (95%CI 0.88–1.16) after harmonization. Because the mean value, + 1.96 × SD, was the upper limit of the CI, the SD before harmonization was 0.31, thus yielding a CV of 21%. The SD after harmonization was 0.07, thus yielding a CV of 7%. The present study found that GI-PET improved the range of CV_repro_ across 12 PET scanners from 3.83–15.3% to 2.30–10.7%, suggesting that inter-scanner variability was improved equally well by GI-PET and EQ.PET. Harmonization is thought to decrease differences in variations among acquisition protocols (2D, 3D, TOF, etc.), machine specificities (such as type of crystal) and other elements (clock synchronisation, activity preparation and injection). However, their study included 517 patients with tumours who were examined using three PET scanners, whereas we examined a NEMA IEC body phantom using 12 PET scanners. The difference may also be affected by the biological features of tumours and patients. Patients should ideally be clinically assessed using the same scanner. However, patients often undergo PET evaluations with different scanners within the same institution. Multicentre studies usually have different scanners, and thus, therapeutic effects can be assessed using various scanners, and patients are often transferred to other hospitals for advanced specialist care.

Harmonization of the SUV also improved the CV_repeat_ of SUV_max_ across 15 frames in each scanner. Velasquez et al. evaluated SUV repeatability in a study of ^18^F-FDG PET and found that the intra-subject CV for SUV_max_ was 10.7% [33]. Doot et al. evaluated the influence of the FWHM of a GF on the repeatability of phantom data and found that increase of smoothing parameter decreased the standard deviation (SD) of the RC [34]. Kelly et al. evaluated the effects of harmonization on quantitative variation in the SUV_max_ relative to the reconstruction protocol. They found that the CV of the SUV_max_ across 15 repeat scans pre-harmonization was 2.81%, 3.25% and 4.69% for OSEM, OSEM+PSF and OSEM+PSF+ time of flight (TOF), respectively, whereas those values after harmonization were 2.28%, 2.00% and 2.58%, respectively [31]. Harmonization is considered to improve the repeatability of any reconstruction protocol.

The CV_repeat_ of all scanners and spheres was < 10% except for that with the 10-mm sphere using the DSTE. With respect to CV repeatability, Lodge et al. reported that the within-subject CV of a tumour SUV was 10% when acquired with careful attention to the protocol [35]. Data acquisition with the DSTE for clinical examinations requires 3 min per bed position, whereas our phantom study acquired data for 2 min per frame in the evaluation of repeatability. This might be why the CV_repeat_ of the 10-mm sphere using DSTE was > 10%.

The SUV_max_ of all PET scanners in the present study was above the lower limit of the reference SUV range. Lasnon et al. examined the RC using Biograph TrueV and found that the RC for a 10-mm sphere on OSEM images was slightly below the lower limit of the EANM proposed range [15]. Harmonization using a GF is available when the RC is above the lower limit of the reference range, and it cannot proceed when the RC is below this limit. However, since some older PET and PET/CT findings of low spatial resolution might have an SUV_max_ below the reference range, the reconstruction settings (including reconstruction methods, matrix, pixel size, iterations, subsets and post-filter) should be carefully considered to obtain an RC within the reference range.

Several limitations are associated with the present study. The basic performance of PET/CT has improved in recent years as the technology has progressed. Therefore, the reference range of the SUV might change with future advancements in PET/CT. We only assessed the SUV_max_, although other metrics, such as metabolic tumour volume and total lesion glycolysis, are also useful [36]. The reproducibility and repeatability of new metrics that might be reliable biomarkers should be evaluated. Differences in the voxel size depending on the reconstruction protocol require consideration. GI-PET automatically converts the original voxel size to the isotropic voxel size, which might result in the SUV differing from the value obtained on a proprietary workstation. A vendor-neutral software for SUV harmonization should be able to apply a 3D-GF to original (non-isotropic voxel) data. The present study is evaluated using only the phantom. Therefore, the feasibility of vendor-neutral software in clinical practice requires validation by further clinical studies.

## Conclusions

We harmonized quantitative values among 12 PET scanners using a commercial vendor-neutral software. This harmonization strategy based on the simple down-smoothing method improved the reproducibility and the repeatability of the SUV determined from a hot lesion. Therefore, this software might enable comparison of SUV directly across different scanners and facilitate multicentre oncology PET studies.

## Abbreviations

CV: Coefficient of variation
DICOM: Digital Imaging and Communications in Medicine
DRO: Digital reference object
FDG: Fluorodeoxyglucose
FWHM: Full-width at half maximum
GF: Gaussian filter
IEC: International Electrotechnical Commission
J-Hart study: Japanese SUV Harmonization Technology study
JSNM: Japanese Society of Nuclear Medicine
NEMA: National Electrical Manufacturers Association
OSEM: Ordered subset expectation maximization
PERCIST: PET Response Criteria in Solid Tumours
PET/CT: Positron emission tomography/computed tomography
PSF: Point spread function
QIBA: Quantitative Imaging Biomarker Alliance
RC: Recovery coefficients
RMSE: Root mean square error
ROI: Region of interest
RSNA: Radiological Society of North America
SD: Standard deviation
SUV: Standardised uptake value
TOF: Time of flight
